# Leveraging Prehospital Care to Address Elder Mistreatment: Development of an Online Training for EMS

**DOI:** 10.1093/geroni/igaa057.705

**Published:** 2020-12-16

**Authors:** Kristin Lees Haggerty, Melanie Miller, Dana Wardlaw, Athi Myint-U, Randi Campetti, Brad Cannell, Kristin Lees

**Affiliations:** 1 Education Development Center, Waltham, Massachusetts, United States; 2 Education Development Center, Waltham, United States; 3 University of Texas Health Science Center at Houston School of Public Health, Dallas, Texas, United States

## Abstract

Elder abuse affects an estimated 1 in 10 older adults in the US and has devastating consequences for their health and well-being yet is widely under-recognized. Prehospital emergency medical service (EMS) providers are particularly well-positioned to identify older adults who are at risk of or experiencing mistreatment, and to report and intervene as appropriate. However, many EMS providers across the country lack the training and tools required to facilitate consistent identification and intervention. Recognizing the critical need for easily accessible, comprehensive, and relevant training, Education Development Center received funding from RRF Foundation for Aging to develop and pilot test the Elder Mistreatment EMS Training Curriculum (EM-ETC). The EM-ETC aims to improve identification, referral, and linkage to coordinated care and support services for older adults who are at risk of mistreatment. In this presentation, we will describe our iterative and collaborative process for developing an interactive online training program for EMS providers. The training leverages the unique circumstances of prehospital care to prepare EMS providers to recognize and respond effectively to elder abuse and help them to fulfill state licensing requirements. We will present findings from our formative research comprising consultation with subject matter experts and state offices of EMS education, focus groups with EMS and Adult Protective Services providers, and user testing with EMS providers.

